# Buckling a Semiflexible Polymer Chain under Compression

**DOI:** 10.3390/polym9030099

**Published:** 2017-03-11

**Authors:** Ekaterina Pilyugina, Brad Krajina, Andrew J. Spakowitz, Jay D. Schieber

**Affiliations:** 1 Center for Molecular Study of Condensed Soft Matter, Illinois Institute of Technology, Chicago, IL 60616, USA; kattisse@gmail.com; 2 Department of Chemical and Biological Engineering, Illinois Institute of Technology, Chicago, IL 60616, USA; 3 Department of Chemical Engineering, Stanford University, Stanford, CA 94305, USA; bkrajina@stanford.edu; 4 Department of Applied Physics, Stanford University, Stanford, CA 94305, USA; 5 Department of Materials Science and Engineering, Stanford University, Stanford, CA 94305, USA; 6 Biophysics Program, Stanford University, Stanford, CA 94305, USA; 7 Department of Physics, Illinois Institute of Technology, Chicago, IL 60616, USA; 8 Department of Applied Mathematics, Illinois Institute of Technology, Chicago, IL 60616, USA

**Keywords:** semiflexible polymers, elasticity, fluctuations

## Abstract

Instability and structural transitions arise in many important problems involving dynamics at molecular length scales. Buckling of an elastic rod under a compressive load offers a useful general picture of such a transition. However, the existing theoretical description of buckling is applicable in the load response of macroscopic structures, only when fluctuations can be neglected, whereas membranes, polymer brushes, filaments, and macromolecular chains undergo considerable Brownian fluctuations. We analyze here the buckling of a fluctuating semiflexible polymer experiencing a compressive load. Previous works rely on approximations to the polymer statistics, resulting in a range of predictions for the buckling transition that disagree on whether fluctuations elevate or depress the critical buckling force. In contrast, our theory exploits exact results for the statistical behavior of the worm-like chain model yielding unambiguous predictions about the buckling conditions and nature of the buckling transition. We find that a fluctuating polymer under compressive load requires a larger force to buckle than an elastic rod in the absence of fluctuations. The nature of the buckling transition exhibits a marked change from being distinctly second order in the absence of fluctuations to being a more gradual, compliant transition in the presence of fluctuations. We analyze the thermodynamic contributions throughout the buckling transition to demonstrate that the chain entropy favors the extended state over the buckled state, providing a thermodynamic justification of the elevated buckling force.

## 1. Introduction

The fundamental physics of instability and transition is common to a broad range of physical phenomena. Important examples include phase transitions in thermodynamic systems, structural transitions in elastic objects under deformation, and instabilities in non-linear dynamical systems. Structural transitions in elastic objects are typically analyzed with an emphasis on geometry, (e.g., object size and shape), material properties, and external perturbations (e.g., applied force and torque), with less focus on fluctuations (either mechanical or thermal). The conditions and nature of a thermodynamic phase transition are fundamentally altered by thermal fluctuations, but the thermodynamic limit typically neglects the system size and geometry. Applied and fundamental studies of physical systems are increasingly focused on molecular structures whose size scale is sufficiently small where both geometrical considerations and thermal fluctuations are central to their manipulation and stability. Here, we consider a physical problem that is both practically important and marries both the mechanical contributions and thermal fluctuations that are central to instability in elastic objects and thermodynamic systems at the microscope.

The buckling of elastic objects arises in many practical applications and has a long history of theoretical analysis. The Euler buckling instability of a rigid rod under a compressive load is the simplest example of elastic instability, where the rod buckles if the compression force exceeds a critical value. Filament buckling under compressive force is widely observed in biopolymers, such as actin filaments, microtubules or DNA [1,2], as well as in synthetic systems like carbon nanotubes polymeric fluids [3] and polymer brushes [4,5]. While fluctuations are crucial in these examples, other physics like excluded volume, neglected here, are also important.

Whereas filament extension is a relatively common experiment, filament buckling experiments are more difficult to conduct and, therefore, are more rare [1,2,6,7,8]. There, the critical buckling force is usually analyzed using classical elasticity theory [9], which ignores fluctuations or is valid only in the zero-temperature limit. However, thermal fluctuations should be taken into account, since the forces measured in single-molecule experiments lie in the pico-Newton range, which are comparable to thermal forces. It has been suggested [10] that the sharp (second-order) transition to the buckled state predicted by Euler does not occur when fluctuations are present, resulting in a smooth (higher-order) transition to the buckled state.

Polymers are usually modeled as random walks: one assumes that the polymer is composed of a chain of statistically independent segments, whether the chain is undiluted or diluted [11,12,13,14]. This model is, in a sense, the opposite extreme of the elastic rod. The conformations of the random-walk model are determined completely by entropy, whereas the conformations of the rigid rod are determined by energy. However, a semiflexible polymer chain is an important physical system where both entropy and energy are significant for chain conformations and dynamics. In such systems, one needs a statistical mechanical model that incorporates both elasticity and fluctuations.

There are four possible approaches to study buckling: approximations made with simplifying assumptions [15,16], asymptotic expansions [17,18,19], numerical simulations, or exact calculations. Without exact results, it can be difficult to assess the reliability of approaches relying on simplifying assumptions. Not surprisingly, then, contradictory results are obtained [10,16,20]. Asymptotic results are reliable but give useful results over a limited domain only, since one must guess the appropriate base case for expansion.

We consider here exact predictions of buckling and of conformations of fluctuating semiflexible polymers—accompanied by Monte Carlo simulations, which provide a helpful visualization. The simulations also provide a strong check of our analytic results, since the two approaches are very different. Surprisingly, we find that fluctuations can be ignored only for very short chains—when the chain length is less than approximately one percent of its persistence length, $l_{p}$ (defined below). We also show that fluctuations stabilize semiflexible polymer to buckling, in contradiction to approximate approaches. Our results can be explained by analyzing thermodynamic contributions to critical buckling force.

## 2. Results

Our starting point is the worm-like chain model [21,22], where the chain is represented as an inextensible space-curve r(s) with arclength variable *s* that runs from 0 to *L*. The deformation of the chain is opposed by a quadratic bending energy

(1) $$\begin{array}{c} E_{B}=\frac{\kappa }{2}\int_{0}^{L}\;\;\;ds{\left(\frac{\partial u}{\partial s}\right)}^{\;\;2}\;, \end{array}$$

where *κ* is the elastic modulus, *L* is the contour length of the chain, $u\left(s\right)=\frac{\partial r}{\partial s}$ is the tangent vector of r(s), and inextensibility is enforced by the constraint $\left|u(s)\right|=1$ at all *s*. In this work, we consider the response of a chain to a compressive force *f*, which is captured by a force energy $E_{f}=f\left|r(L)-r(0)\right|=fR$ that favors having the ends in close spatial proximity.

We define the persistence length $l_{p}=\frac{\kappa }{k_{B}T}$ as the length of chain over which a curved conformation (with local curvature $1/l_{p}$) has an energy comparable to $k_{B}T$. Predictions using the worm-like chain model represent the thermal behavior of a bendable but unstretchable elastic thread, and the behavior reduces to that of a classical elastic chain in the limit of zero temperature (or large persistence length $l_{p}$). At finite temperature, the persistence length $l_{p}$ defines the length scale of correlation between tangent vectors along the chain.

The bending energy $E_{B}$ provides the necessary input to predict the minimum-energy shape of a chain. Appendix A provides an overview of the buckling of an elastic filament [9], resulting in a theoretical prediction of the critical buckling force $f_{E}$ (or the Euler buckling force) and the shape of the minimum-energy conformation. The critical Euler force $f_{E}$ depends on the rod length *L* and persistence length $l_{p}$ as $f_{E}=k_{B}Tl_{p}\pi^{2}/L^{2}$. For forces below $f_{E}$, the minimum-energy conformation is a straight chain with R=L, and at larger forces, the chain adopts a bent (or buckled) conformation.

A thermally fluctuating elastic filament (i.e., a worm-like chain) is governed by the Green function G(R;L), which gives the probability that the end-to-end distance is given by *R* and is formally found by path integration over all conformations u(s) to be

(2) $$G\left(R;L\right)=\int \;Du\left(s\right)\delta \;\left(\;R-\left|\int_{0}^{L}\;\;\;dsu\left(s\right)\right|\right)\exp\;\left(-\frac{E_{B}\left[u(s)\right]}{k_{B}T}\right).$$

In this work, we exploit a method that calculates the exact Green function G(K;p) for the worm-like chain model, which is the Fourier–Laplace transform of G(R;L) (Fourier transform R→K and Laplace transform L→p). The Green function G(K;p) is written in the continued fraction representation [23,24]

(3) $$\begin{array}{c} G\left(K;p\right)=\frac{1}{P_{0}+\frac{{\left(a_{1}K\right)}^{2}}{P_{1}+\frac{{\left(a_{2}K\right)}^{2}}{P_{2}+\frac{{\left(a_{3}K\right)}^{2}}{\cdots }}}}, \end{array}$$

where $P_{l}=p+l\left(l+1\right)$, and $a_{l}=l/\sqrt{\left(2l+1)(2l-1\right)}$. To use Equation (3), we must inverse Laplace transform from *p* to the dimensionless chain length $N=L/(2l_{p})$ and inverse Fourier transform from *K* to dimensionless end separation $r=R/(2l_{p})$. Our previous work [23,24] provides efficient methods to perform these inversions, and we provide numerical realizations of the Green function G(R;L) on our research group websites [25,26].

The Helmholtz free energy of a worm-like chain subjected to a compressive force *f* is given by

(4) $$\begin{array}{c} \frac{F(R,L)}{k_{B}T}=-\log\;\left[R^{2}G\left(R;L\right)\right]+fR. \end{array}$$

**Figure 1:**
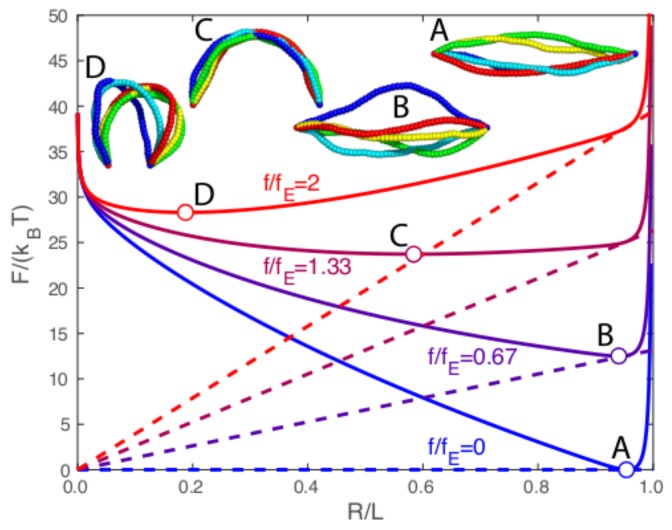
Free energy *F* as a function of end-to-end separation over contour length R/L for a semiflexible chain of length $L/(2l_{p})=0.25$ at four different applied forces $f/f_{E}$. Solid curves show the total free energy *F*, and the dashed lines indicate the force energy $E_{f}$. Monte Carlo snapshots of the chain conformations at the free-energy minima are marked by the circles labeled *A*, *B*, *C*, and *D*.

Figure 1 shows the free energy *F* versus the end extension *R* for a chain length of $L/(2l_{p})=1/4$ at four values of the force *f*, ranging from $f/f_{E}=0$ to $f/f_{E}=2$. This range of forces corresponds to two forces below the zero-temperature critical force $f_{E}$ and two forces above. The solid curves are the total free energy *F*, and the dashed curves indicate the force energy $E_{f}=fR$ at each force. On each curve, the circle indicates the location of the free-energy minimum.

Figure 1 provides a clear indication of the non-linear response of a worm-like chain to a compressive force. Between $f/f_{E}=0$ and $f/f_{E}=0.67$, there is very little change in the minimum free-energy end extension $R_{\min}$, indicating a large resistance to compressive force. However, between $f/f_{E}=0.67$ and $f/f_{E}=1.33$, there is considerable end retraction associated with a dramatic compliance in this force range. We emphasize that all of the conformation images exhibit dramatic conformational fluctuations, despite the fact that the chain length $L/(2l_{p})=0.25$ is significantly shorter than a persistence length.

**Figure 2:**
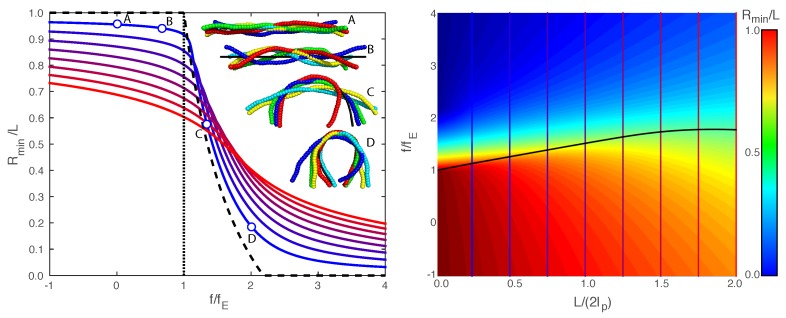
The left side shows the minimum free-energy end extension $R_{\min}$ versus external force $f/f_{E}$ for values of $L/(2l_{p})$ ranging from $L/(2l_{p})=0.25$ (blue curve) to $L/(2l_{p})=2$ (red curve). For $L/(2l_{p})=0.25$, the top plot includes pre-buckling (marked *A* and *B*) and post-buckling (marked *C* and *D*) conformations calculated via Monte Carlo simulation at the same force values as in Figure 1. The right side shows a surface plot of $R_{\min}$ ($R_{\min}/L=1$ in red to $R_{\min}/L=0$ in blue as indicated by the colorbar) versus $f/f_{E}$ and $L/(2l_{p})$. The vertical lines indicate the $L/(2l_{p})$-value slices that are plotted in the top plot. The black curve on the bottom surface plot gives the critical buckling force made dimensionless by the zero-temperature critical force $f_{c}/f_{E}$ versus $L/(2l_{p})$.

Figure 1 also provides a procedure for mapping out the minimum free-energy end extension $R_{\min}$ versus the compressive force *f* for a fixed chain length, shown in Figure 2. The left side of Figure 2 shows $R_{\min}$ versus *f* for chain lengths ranging from $L/(2l_{p})=0.25$ (blue curve) to $L/(2l_{p})=2$ (red curve). The compressive force ranges from $f/f_{E}=-1$ to $f/f_{E}=4$, where negative values correspond to a worm-like chain under tension. The dashed curve in Figure 2 shows the zero-temperature (i.e., no fluctuations) end extension [9], and the dotted vertical line gives the zero-temperature critical force $f=f_{E}$. The inset images show realizations from Monte Carlo simulations at the same four force values as in Figure 1. We note that the Monte Carlo images in Figure 1 have a fixed end extension given by $R_{\min}$, whereas the Monte Carlo images in Figure 2 are from simulations with a fixed force *f*, and the ends undergo thermal fluctuations as dictated by the shape of the free energy curve. The large fluctuations of chain conformations seen at points C and D in Figure 2 are not surprising, given how flat the free energy curves are for C and D in Figure 1.

The zero-temperature end extension (dashed line in Figure 2) indicates a second-order transition at the critical force $f_{E}$. In contrast, the chain lengths with finite temperature exhibit a smooth transition for forces near the zero-temperature critical force $f_{E}$. We interpret buckling of a thermally fluctuating chain as having a nonlinear compliance or a “softening” of the response of the end extension to compressive force. We define a response function $\alpha_{F}\equiv -\frac{dR_{\min}}{df}$, and we define the critical force $f_{c}$ as the force with the largest response in $R_{\min}$ versus force *f* (i.e., a maximum value in the response function $\alpha_{F}$). In other words, the critical force $f_{c}$ corresponds to an inflection point on the $R_{\min}$ versus *f* curve.

The left side of Figure 2 provides constant-*L* curves of $R_{\min}$ versus *f*, so this information can be represented by a surface plot of $R_{\min}$ versus *f* and $L/(2l_{p})$. The right side of Figure 2 shows a bird’s-eye view of this surface plot with red indicating $R_{\min}=1$ and blue corresponding to $R_{\min}=0$. The colored vertical lines indicate the $L/(2l_{p})$ values plotted on the left side of Figure 2. The black curve shows the critical force $f_{c}$ for each $L/(2l_{p})$ value. One sees that fluctuations actually enhance the stability of the chain to buckling by as much as 80%. As the length of the chain goes to zero, the force naturally converges to the zero-temperature or the Euler-buckling limit.

**Figure 3:**
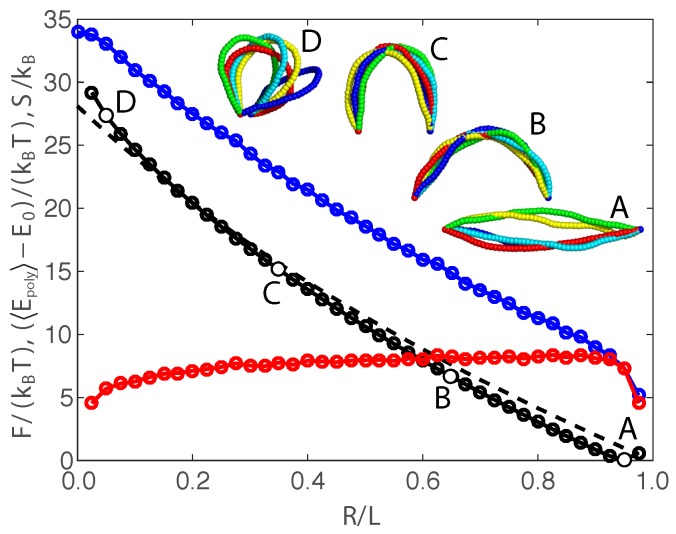
Thermodynamic contributions to the free energy *F* for a worm-like chain versus the end-to-end distance *R*. The free energy *F* (black dots) is determined using our exact analytical treatment (Equation (4)). The average polymer energy $\langle E_{\mathrm{poly}}\rangle -E_{0}$ (blue dots) is found from discretized Monte Carlo simulations, and the entropy *S* (red dots) is calculated from $S=(\langle E_{\mathrm{poly}}\rangle -E_{0}-F)/T$. The dashed curve is the zero-temperature bending energy, and the four Monte Carlo conformations span the range of end retractions.

The counterintuitive behavior of the critical force $f_{c}$ versus the length $L/(2l_{p})$ can be explained by parsing out the thermodynamic contributions to the free energy. To determine each thermodynamic contribution, we exploit our Monte Carlo simulations with finite degrees of freedom (Appendix B). The average polymer energy $\langle E_{\mathrm{poly}}\rangle -E_{0}$ converges in the limit of large bead number, and the entropy is found using $S=(\langle E_{\mathrm{poly}}\rangle -E_{0}-F)/T$. The reference energy $E_{0}$ defines a thermodynamic energy that arises from the discretization of the polymer chain. The value of the reference energy is $E_{0}=\frac{5}{2}k_{B}T\left(N_{b}-4\right)$, where $N_{b}$ is the number of beads in the discretized chain. This value offsets the thermodynamic contribution from the energy associated with the equipartition theorem for each active mode in the discrete chain (see Appendix B for more details). Figure 3 gives results from a Monte Carlo simulation for $N_{b}=51$ beads, and Appendix B demonstrates that these results are invariant to the discretization.

Figure 3 shows the free energy *F* (black dots), average bending energy $\langle E_{\mathrm{poly}}\rangle -E_{0}$ (blue dots), and entropy TS (red dots) versus end retraction for a worm-like chain with length $L/(2l_{p})=1/4$ and zero compressive force *f*. The four Monte Carlo snapshots indicate typical conformations over the range of end retraction. The dashed curve gives the polymer energy $E_{\mathrm{poly}}$ in the zero-temperature limit. For this relatively short chain ($L=l_{p}/2$), the free energy *F* closely tracks with the zero-temperature bending energy, indicating that the response is largely dictated by the bending energy. However, there is a systematic trend of faster increase of the free energy *F* with end retraction than the zero-temperature behavior. This observation is consistent with the elevation of the critical buckling force with length *L* or, equivalently, with the degree of thermal fluctuations.

This subtle trend is dictated by the entropic contribution. The free-energy minimum coincides with the maximum-entropy state, and the entropy *S* exhibits a mild decrease with the decreasing *R*. Thus, the extended state is both energetically favored and entropically favored, and the critical force is, therefore, larger than the zero-temperature Euler critical force $f_{E}$. We note that the entropic favorability arises from the conformational fluctuations that are accessible at each end retraction. This should not be confused with the entropic preference for a flexible Gaussian chain to have zero end-to-end distance, since a flexible chain does not exclude conformations based on bending deformation.

## 3. Discussion

In this work, we determine that fluctuations can be ignored only for chains much shorter than their persistence length. In fact, we find that the buckling phenomenon is never purely elastic for macromolecules, except on length scales where the granularity of monomers becomes important, and a continuous rod is a poor assumption. Our exact results show unambiguously that fluctuations indeed stabilize semiflexible chains to buckling in 3D. The counterintuitive behavior of the critical force occurs because the extended state is thermodynamically—both energetically and entropically—favored at the critical region, and the critical force needed to overcome both thermodynamic contributions is elevated above the zero-temperature critical force $f_{E}$. Our exact calculations provide a strong check for numerical simulations and testable hypotheses for experimental phenomena.

Many critical biological processes involve deformation of semiflexible biopolymers such as DNA and filamentous proteins. The formation of gene regulatory complexes involve looping segments of DNA to bind to protein complexes. The buckling process involved in looping distal segments of DNA is controlled by the elastic mechanics of the DNA strand and the entropic change upon docking the DNA strand into the regulatory complex [27,28]. Single-molecular measurements of the kinetics of DNA binding to Lac repressor for DNA lengths of approximately 100 basepairs (i.e., $L/(2l_{p})\approx 0.67$) demonstrate that the time scale associated with loop formation is highly sensitive to DNA mechanics (both bending and twisting), leading to time scales around 100 seconds for DNA binding [27]. Our theoretical predictions based on the worm-like chain model for the kinetics of loop formation and loop dissociation exhibit quantitative agreement with the measured in vitro binding and unbinding time scales [27,28]. The in vivo forces involved in loop formation in this regulatory complex arise from protein physical interactions (e.g., binding proteins including Lac repressor and Catabolite Activator protein), motor protein activity, and thermal fluctuations. Our current work provides a physical basis for interpreting the impact of conformational fluctuations in modulating the forces necessary to induce a biopolymer to adopt such a buckled conformation.

Single-molecule measurements have been instrumental in elucidating the response of individual biopolymers to external forces and torques [29,30,31,32,33]. A closely related problem to the Euler buckling phenomenon considered in this manuscript is the response of an elastic filament to externally applied torque [9,34]. For an elastic filament under tension and subjected to torque, elastic strain associated with twist deformation leads in a buckling transition at a critical torque, resulting in the formation of a looped “hackle” in the filament. The hackle causes an abrupt decrease in the end-to-end distance of the filament. This phenomenon is observed in DNA strands subjected to external torque using an angular optical optical trap [32]. However, the experimentally observed end-to-end retraction [32] exceeds predictions from classical elasticity theory [9,34]. This effect is modulated by salt concentration [33], indicating that self repulsion plays a non-trivial role in the twist induced buckling transition. In contrast to Euler buckling, which exhibits a distributed buckle over the length of the polymer, twist-induced buckling localizes the instability within a small loop that would have considerable self-interaction. However, the impact of conformational fluctuations is an important contribution in the twist buckling phenomenon, and our work on the statistical behavior of the worm-like chain model with twist [35] would serve as a valuable starting point for performing analogous predictions as presented in this work.

Current single-molecule experimental methods primarily apply tension to measure mechanical properties of biopolymers and mechanochemical behavior in enzymes [30,31]. In order to experimentally test the predictions in this manuscript, it would be necessary to develop a single-molecule apparatus or a DNA construct that either directly applies a compressive force or is capable of transducing a tensile force to a compressive force. One approach that could be developed is to construct a DNA or RNA nanostructure that can be pulled in one direction, resulting in a compression in another direction. For example, a cube structure can be pulled at opposite vertices, resulting in elongation along the inter-vertex axis and compression along the orthogonal axes. More complicated architectures resulting in tensegrity structures [36] could be exploited in determining biopolymer response using a tensile apparatus. Furthermore, such structures are proposed as a structural motif in cellular mechanics [36] with complex elastic properties, and the study of such structures would be valuable for their intrinsic nonlinear elastic properties. The impact of thermal fluctuations on the response of biopolymer structures and networks is crucial in a variety of biological phenomena, and our work serves to address the buckling phenomenon within the individual segments of a more complex molecular architecture.

It follows that the role of thermal fluctuations for buckling phenomena cannot be neglected during the analysis of collective compression-resistant behavior of, for example, actin networks or nanotubes [37]. Understanding of collective behavior or network response to deformations starts with the simpler problem of how a single semiflexible polymer responds to compressive force. We hope that our findings will encourage experimentalists to seek reliable data on critical buckling force for a single semiflexible filament. It would be interesting to study whether fluctuations generally stabilize transitions in other microscopic systems. The work presented here presents a roadmap for attacking such problems.

## Figures and Tables

**Figure A1:**
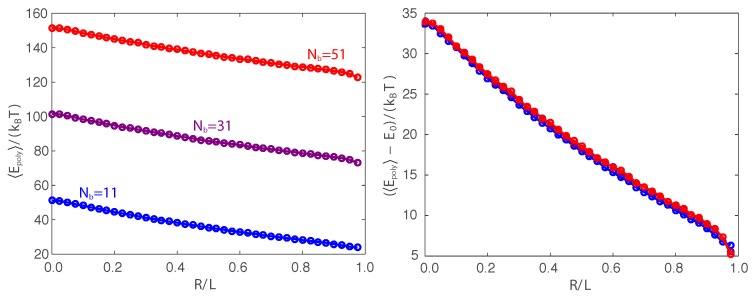
The left plot shows the average polymer energy $\langle E_{\mathrm{poly}}\rangle$ versus the end extension from Monte Carlo simulations for a chain of length $L=l_{p}/2$ and number of beads $N_{b}=11$ (blue), $N_{b}=31$ (purple), and $N_{b}=51$ (red). The right plot demonstrates that subtracting the references’ energy $E_{0}=\frac{5}{2}k_{B}T\left(N_{b}-4\right)$ from each average polymer energy $\langle E_{\mathrm{poly}}\rangle$ gives an average polymer energy that is invariant to the discretization and reflects the deformation energy of the fluctuating polymer chain under compression.

## Appendix A. Euler Buckling in the Zero-Temperature Limit

At zero temperature, the polymer shape is given by the minimum-energy conformation, which is found by setting the first variation of the energy to zero. The energy of an elastic filament with orientation given by tangent vector u(s), or, equivalently, by polar coordinates in a given coordinate system θ(s), ϕ(s) is written as

(A1) $$\frac{E_{B}\left(\theta \left(s\right),\phi \left(s\right)\right)}{k_{B}T}=\frac{l_{p}}{2}\int_{0}^{L}\;\;\;ds\left[{\left(\frac{\partial \theta }{\partial s}\right)}^{\;2}+\sin^{2}\theta {\left(\frac{\partial \phi }{\partial s}\right)}^{\;2}\right]+\beta fR.$$

The first variation (or differential change) of the end-to-end distance *R* is written as

(A2) $$\delta R=\frac{\vec{R}}{R}\cdot \delta \vec{R}.$$

Now, we align the end-to-end vector to the *z*-axis, such that $\vec{R}/R=\hat{z}$. The first variation in the energy is now written as

(A3) $$\begin{array}{ccc} \delta \beta E_{B} & = & l_{p}\int_{0}^{L}\;\;\;ds\left[\frac{\partial \theta }{\partial s}\frac{\partial \delta \theta }{\partial s}+\sin\theta \cos\theta {\left(\frac{\partial \phi }{\partial s}\right)}^{2}\delta \theta +\sin^{2}\theta \frac{\partial \phi }{\partial s}\frac{\partial \delta \phi }{\partial s}\right]+\beta f\hat{z}\cdot \delta \vec{R}\\ & = & l_{p}\int_{0}^{L}\;\;\;ds\left[\frac{\partial \theta }{\partial s}\frac{\partial \delta \theta }{\partial s}+\sin\theta \cos\theta {\left(\frac{\partial \phi }{\partial s}\right)}^{2}\delta \theta +\sin^{2}\theta \frac{\partial \phi }{\partial s}\frac{\partial \delta \phi }{\partial s}\right]-\beta f\int_{0}^{L}\;\;\;ds\sin\theta \delta \theta \\ & = & -\int_{0}^{L}\;\;\;ds\left[l_{p}\frac{\partial^{2}\theta }{\partial s^{2}}-l_{p}\sin\theta \cos\theta {\left(\frac{\partial \phi }{\partial s}\right)}^{2}+\beta f\sin\theta \right]\delta \theta +\\ & & -l_{p}\int_{0}^{L}\;\;\;ds\frac{\partial }{\partial s}\left(\sin^{2}\theta \frac{\partial \phi }{\partial s}\right)\delta \phi +{\left.l_{p}\frac{\partial \theta }{\partial s}\delta \theta \right|}_{0}^{L}+{\left.l_{p}\sin^{2}\theta \frac{\partial \phi }{\partial s}\delta \phi \right|}_{0}^{L}, \end{array}$$

where we performed integration by parts to obtain the last line. This gives two coupled Euler-lagrange equations

(A4) $$\begin{array}{ccc} l_{p}\frac{\partial^{2}\theta }{\partial s^{2}}-l_{p}\sin\theta \cos\theta {\left(\frac{\partial \phi }{\partial s}\right)}^{2}+\beta f\sin\theta & = & 0, \end{array}$$

(A5) $$\begin{array}{ccc} \frac{\partial }{\partial s}\left(\sin^{2}\theta \frac{\partial \phi }{\partial s}\right) & = & 0, \end{array}$$

which is subject to the boundary conditions

(A6) $$\begin{array}{ccc} & & \frac{\partial \theta (0)}{\partial s}=\frac{\partial \theta (L)}{\partial s}=0, \end{array}$$

(A7) $$\begin{array}{ccc} & & \sin^{2}\theta \left(0\right)\frac{\partial \phi (0)}{\partial s}=\sin^{2}\theta \left(L\right)\frac{\partial \phi (L)}{\partial s}=0. \end{array}$$

The solution to the 2nd Euler-Lagrange equation is $\phi =\phi_{0}$, which simplifies the 1st Euler-Lagrange equation

(A8) $$l_{p}\frac{\partial^{2}\theta }{\partial s^{2}}+\beta f\sin\theta =0.$$

The Euler–Lagrange equation is solved using a symmetric formulation. Define σ=2s/L-1, resulting in the dimensionless Euler–Lagrange equation

(A9) $$\frac{\partial^{2}\theta }{\partial \sigma^{2}}+\frac{\tilde{f}}{4}\sin\theta =0,$$

where $\tilde{f}=\beta fL^{2}/l_{p}$. The boundary conditions in the new variables are θ(σ=0)=0 and $\frac{\partial \theta (\sigma =1)}{\partial \sigma }=0$. The solution to the Euler–Lagrange equation for σ∈[-1,0] is just the negative of the solution from σ∈[0,1]. The mathematical form of *θ* is written as

(A10) $$\theta =2\;\mathrm{am}\left(\left.\sigma \sqrt{1-\cos\theta_{f}}\frac{\sqrt{F}}{2\sqrt{2}}\right|\csc^{2}\left(\theta_{f}/2\right)\right),$$

where am(x|m) is the Jacobi amplitude, which is the inverse function of the elliptic integral

(A11) $$F\left(\psi |m\right)=\int_{0}^{\psi }d\psi^{\prime }{\left(1-m\sin^{2}\psi^{\prime }\right)}^{-1/2}.$$

The final orientation $\theta_{f}$ is the angle at σ=1, which is found from the transcendental equation

(A12) $$\theta_{f}=2\;\mathrm{am}\left(\left.\sqrt{1-\cos\theta_{f}}\frac{\sqrt{F}}{2\sqrt{2}}\right|\csc^{2}\left(\theta_{f}/2\right)\right).$$

There is always a solution θ=0. However, θ≠0 arises for forces above a critical value of ${\tilde{f}}_{c}=\pi^{2}$. Numerical solution of Equation (A12) is used to find $\theta_{f}$, and the filament shape is determine by subsequent evaluation of Equation (A10).

## Appendix B. Monte Carlo Simulations

We perform Monte Carlo simulations of a worm-like chain under compressive force. The polymer chain is modeled as a thermally fluctuating isotropic elastic filament whose conformations are dictated by a polymer chain energy $E_{\mathrm{poly}}$. The inextensible worm-like chain model is defined to be a continuous chain, and implementation of this model into a simulation requires discretization of the chain to $N_{b}$ beads. In our previous work [38,39], we demonstrate that the process of discretizing the chain results in quadratic-order deformation modes that go beyond the bare bending energy of the continuous chain. A simple example of this effect is that the continuous inextensible worm-like chain model has an infinite chain stretching modulus; however, a discretized worm-like chain would manifest a stretching mode due to the entropic elasticity from the implicit chain between the beads. At the quadratic level, the discrete polymer chain energy contains a bending, stretching, shearing, and bend-shear coupling that accounts for the underlying thermal fluctuations of the continuous chain [38,39].

The resulting discrete, coarse-grained model for an inextensible worm-like chain utilizes our discrete shearable stretchable worm-like chain (dssWLC) [38,39] with $N_{b}$ discrete beads. The dssWLC defines a universal coarse-graining procedure that is able to accurately capture chain statistics at all length-scales above the discretization Δ [38,39]. In contrast to the bare discrete worm-like chain, the polymer configuration is additionally represented by an auxiliary set of orientation vectors ${\vec{u}}_{i}$, which capture the coarse-grained tangent of the chain configuration in the vicinity of ${\vec{r}}_{i}$. At a given length-scale of coarse-graining Δ, the conformational free energy of the chain is dictated by six effective elastic parameters that capture the conformational statistics at the level of coarse-graining. The bending modulus $\epsilon_{B}$ elastically couples adjacent local chain orientation ${\vec{u}}_{i}$,${\vec{u}}_{i-1}$. The stretch modulus $\epsilon_{∥}$ characterizes the entropic cost of stretching the chain away from its ground-state extension *γ*. The shear modulus $\epsilon_{⊥}$ penalizes shearing of the local chain orientation away from the inter-bead displacement vector ${\vec{R}}_{i}={\vec{r}}_{i}-{\vec{r}}_{i-1}$. The bend-shear coupling modulus *η* characterizes the tendency of the chain to shear in the same direction as bending. The chain conformational free energy is given by

(A13) $$E_{\mathrm{poly}}=\sum_{i=2}^{N_{b}}\left[\frac{\epsilon_{b}}{2\Delta }|{\vec{u}}_{i}-{\vec{u}}_{i-1}-\eta {\vec{R}}^{⊥}{|}^{2}+\frac{\epsilon_{∥}}{2\Delta }{\left({\vec{R}}_{i}\cdot {\vec{u}}_{i-1}-\Delta \gamma \right)}^{2}+\frac{\epsilon_{⊥}}{2\Delta }{\left|{\vec{R}}_{i}^{⊥}\right|}^{2}\right],$$

where ${\vec{R}}_{i}^{⊥}={\vec{R}}_{i}-{\vec{R}}_{i}\cdot {\vec{u}}_{i-1}$ is the component of the inter-bead displacement vector perpendicular to ${\vec{u}}_{i-1}$. Our research group website [25] contains Matlab (R2017a, MathWorks, Natick, MA, USA) scripts to determine the elastic parameters for the dssWLC model and for performing Monte Carlo and Brownian dynamics simulations of this model.

The average polymer chain energy $\langle E_{\mathrm{poly}}\rangle$ cannot be determined directly from the continuous worm-like chain free energy *F*, given by Equation (4). The formulation of the worm-like chain represents the polymer as a continuous elastic thread, having infinite degrees of freedom. Equipartition theorem dictates that each thermally active degree of freedom would contribute $k_{B}T/2$ to the average energy and $k_{B}/2$ to the entropy. Thus, the worm-like chain model predicts infinite average energy and infinite average entropy, which results in several unphysical effects such as diverging polymer stress in the continuous-chain limit [40]. Although each contribution independently diverges, the free energy *F* is finite and well defined, and our exact results provide this thermodynamic contribution.

Our Monte Carlo simulations provide a framework to evaluate the change in the average polymer chain energy $\langle E_{\mathrm{poly}}\rangle$ with end extension, provided we can reliably translate the discrete chain behavior to the continuous limit. We perform several simulations with an increasing number of beads to determine the average polymer chain energy $\langle E_{\mathrm{poly}}\rangle$. The left plot of Figure A1 shows results from Monte Carlo simulations of a polymer chain of length $L=l_{p}/2$ with bead number $N_{b}=11$ (blue), $N_{b}=31$ (purple), and $N_{b}=51$ (red). These results demonstrate the increase in the average polymer chain energy with the number of beads, reflecting the divergence in the energy as the number of degrees of freedom increases. However, the thermodynamic contribution from these active modes is easily interpreted and eliminated from these results. Since each bead has a position and orientation, there are five degrees of freedom per bead. Thus, a free chain has an average polymer chain energy predicted from the equipartition theorem to be $\frac{5}{2}k_{B}T\left(N_{b}-1\right)$. We define a reference energy $E_{0}=\frac{5}{2}k_{B}T\left(N_{b}-4\right)$ that includes the free-chain energy in order to eliminate the contribution from discretization; the factor of four is a convenient choice for visualization of the results in Figure 3. The right plot of Figure A1 shows our simulation results for average polymer chain energy $\langle E_{\mathrm{poly}}\rangle -E_{0}$ (i.e., subtracting the reference energy $E_{0}$) for bead number $N_{b}=11$ (blue), $N_{b}=31$ (purple), and $N_{b}=51$ (red). The coarsest discretization ($N_{b}=11$, blue) exhibits slight numerical deviations from the results from simulations with $N_{b}=31$ (purple) and $N_{b}=51$ (red). With increasing $N_{b}$, the average polymer energy $\langle E_{\mathrm{poly}}\rangle -E_{0}$ is invariant to the number of beads $N_{b}$, and we can interpret the average polymer energy $\langle E_{\mathrm{poly}}\rangle -E_{0}$ as the thermalized deformation energy of a buckled polymer chain. The entropy *S* is then found from our exact results for the free energy *F* and the average polymer energy $\langle E_{\mathrm{poly}}\rangle -E_{0}$ using the thermodynamic expression $S=(\langle E_{\mathrm{poly}}\rangle -E_{0}-F)/T$. Our results for these thermodynamic contributions based on Monte Carlo simulations with $N_{b}=51$ are shown in Figure 3.
